# Peroneal tendon irritation after arthroscopic modified Broström procedure: A case report

**DOI:** 10.1097/MD.0000000000018424

**Published:** 2019-12-20

**Authors:** Young Koo Lee, Hong Seop Lee, Whi Je Cho, Sung Hun Won, Chang Hyun Kim, Hyun Kwon Kim, Aeli Ryu, Woo Jong Kim

**Affiliations:** a Department of Orthopaedic Surgery, Soonchunhyang University Hospital Bucheon, Wonmi-gu, Bucheon; b Department of Foot and Ankle Surgery, Nowon Eulji Medical Center, Eulji University, Nowon-gu; c Department of Orthopaedic Surgery, Soonchunhyang University Hospital Seoul, Yongsan-gu, Seoul; d Department of Obstetrics and Gynecology; e Department of Orthopaedic Surgery, Soonchunhyang University Hospital Cheonan, Dongam-gu, Cheonan, Korea.

**Keywords:** arthroscopic modified Broström procedure, peroneal tendon irritation, suture knot irritation

## Abstract

**Rationale:**

With the development of ankle arthroscope techniques and procedures, the number of arthroscopic modified Broström procedures (MBPs) is increasing. All-inside arthroscopic MBP was developed recently, with good to excellent results. However, several complications have been reported in patients after arthroscopic MBP. This case report describes a rare complication of arthroscopic MBP.

**Patient concerns:**

A 34-year-old woman presented with severe pain in her right ankle and underwent arthroscopic MBP for lateral ankle instability. About 6 months postoperatively, she presented with severe pain on the lateral aspect of the right ankle, especially while walking.

**Diagnosis:**

In physical examinations, there was marked swelling around the ankle and focal tenderness in the posterolateral malleolar area. Ankle ultrasonography showed a diffuse low-echoic mass-like lesion at the distal fibula between the fibular tip and peroneus tendon. T1-weighted sagittal magnetic resonance imaging images showed an irregularly shaped mass-like lesion with a heterogeneous signal near the distal fibula posteriorly where the anchor protruded.

**Interventions:**

The suture anchor in the posterior distal fibula area, which had irritated the peroneus tendon, was removed with debridement of the granulomatous lesion.

**Outcomes:**

At the 3-month follow-up, the patient was almost asymptomatic and had a nearly full range of motion. No complications or recurrent symptoms were noted at the 1-year follow-up.

**Lessons:**

Three-dimensional computed tomography studies of the appropriate fibular depth and position of suture anchors are needed to standardize the procedure and reduce complications.

## 1 Introduction

Ankle sprains are common sports injuries and may sometimes result in chronic lateral ankle instability.^[1–3]^ Following an acute ankle sprain, nonoperative treatment is recommended initially, including rest, ice, compression, limb elevation, and functional rehabilitation.^[4]^ Surgery is indicated for those patients in whom nonoperative attempts fail.^[5,6]^

In 1966, Broström proposed an anatomic procedure to repair the anterior talofibular ligament (ATFL) using its remnants^[7]^; Gould modified this technique in 1980 by reinforcing the inferior extensor retinaculum,^[8]^ and Karlsson proposed reattaching the ATFL to the fibula at its anatomical origin through drill holes.^[4,9]^ The main advantages of this anatomic technique are the simplicity of the procedure, possibility of restoring the physiological joint anatomy and kinematics, and preservation of subtalar joint mobility.^[7,10]^ After 50 years, the open modified Broström anatomic repair technique is widely accepted as the reference standard for lateral ankle stabilization.^[10,11]^

With developments in ankle arthroscope techniques and procedures, the number of arthroscopic-modified Broström procedures (MBPs) is increasing. The all-inside arthroscopic MBP was developed recently and yields good to excellent results.^[12–15]^ Arthroscopic MBP is technically straightforward,^[16]^ and reduces the recovery time by limiting surgical dissection of the tissues about the ankle joint, while providing comparable ankle stability to open procedures.^[13,15–17]^

However, several complications have been reported in patients after arthroscopic MBP, including sural nerve neuritis, superficial peroneal nerve neuritis, portal site irritation, prominent asymptomatic suture knots, and knot pain.^[4,13,14,18]^

We present a rare case of peroneal tendon irritation caused by the suture anchor after an arthroscopic Broström procedure.

## 2 Case description

This case report was approved by the Institutional Review Board (IRB) of Soonchunhyang University Hospital (IRB No. 2019-05-021). The patient consented to publication of this report and the accompanying images.

A 34-year-old woman with a body mass index of 30.85 kg/m^2^ (height 160 cm, weight 79 kg) presented with severe pain in her right ankle. She had no underlying disease. She had a history of lateral malleolus fracture of the right ankle 6 years earlier and underwent internal fixation with a plate. The internal device was removed 1 year after the fracture and she had no residual pain or other symptoms.

**Figure 1:**
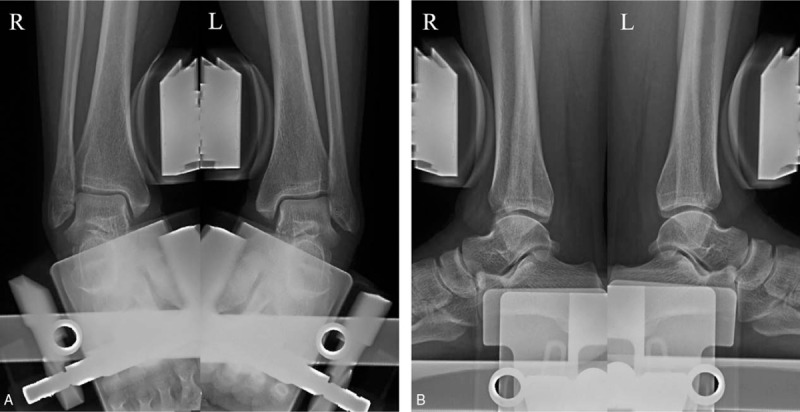
Preoperative anterior drawer test (A) and varus stress view (B) radiographs.

**Figure 2:**
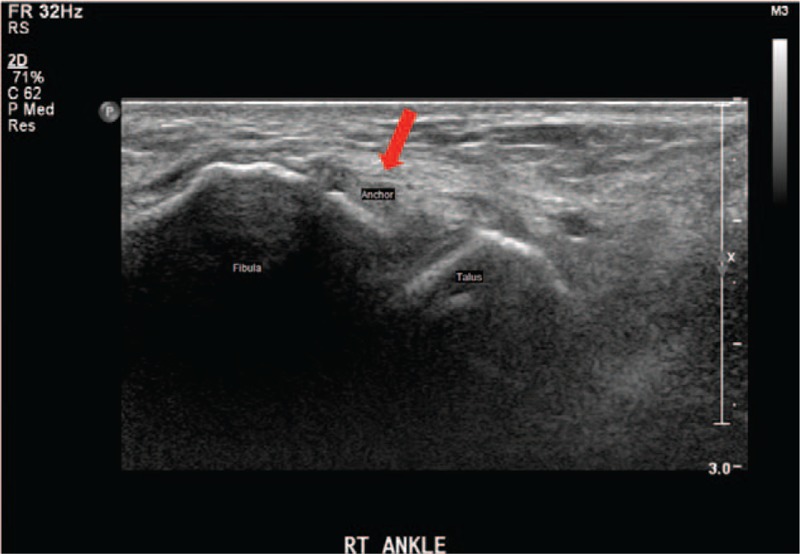
Preoperative ankle ultrasonography shows the suture anchor protruding posteriorly at the fibula tip.

**Figure 3:**
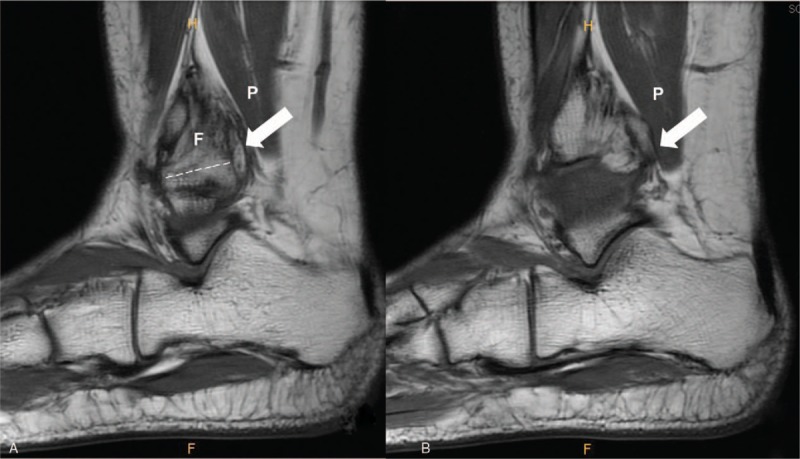
Preoperative magnetic resonance imaging shows the superior suture anchor (dotted line) (A) and mass-like lesion with a heterogeneous signal (white arrow) behind the site of anchor protrusion (B) on T1-weighted sagittal images (F, fibula, P, peroneus tendon).

She had no complaints with her right ankle until an acute ankle sprain 16 months before visiting our hospital. Subsequently, she complained of pain and instability of her right ankle joint. At least once a week, the ankle felt like it had given way while walking. Conservative treatment including medication, physical treatment, and exercise treatment was tried for 4 months, but the pain and sensed instability persisted. With a diagnosis of chronic ankle instability, arthroscopic MBP was performed on her ankle elsewhere 1 year before visiting our clinic. A short-leg cast was applied for 1 month after that surgery, after which rehabilitation was started. Five weeks postoperatively, weight-bearing walking was permitted and she started ordinary activities. She had no ankle pain or discomfort after the arthroscopic MBP. Six months postoperatively, she presented to our clinic with severe pain on the lateral aspect of the right ankle, particularly during motion. Physical examination showed marked swelling around the ankle and focal tenderness in the posterolateral malleolus area. The anterior drawer test was negative and the talar tilt angle was 3.2° on a varus stress view radiograph, which was within the normal range (Fig. 1). Ankle ultrasonography showed a diffuse mass-like low-echoic lesion at the distal fibula between the fibular tip and peroneus tendon (Fig. 2). T1-weighted sagittal magnetic resonance imaging showed an irregular mass-like lesion with heterogeneous signals posterior to the distal fibula, where the anchor protruded (Fig. 3). The Ankle-Hindfoot scale of the American Orthopedic Foot and Ankle Society (AOFAS) score was 74 and a visual analog scale (VAS) score for pain was 8 points.

**Figure 4:**
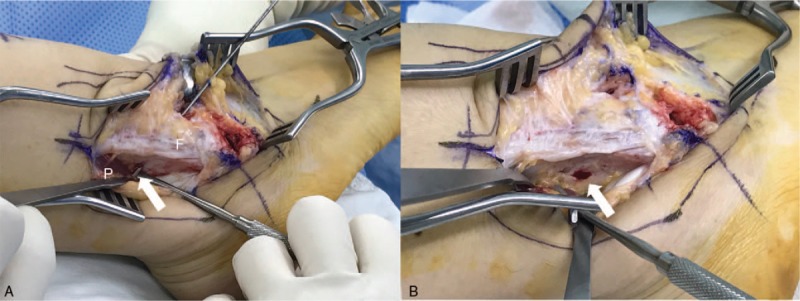
Intraoperative finding images. Granulomatous mass-like lesion around the peroneus tendon at the site of superior anchor protrusion (arrow) (A), and removal of the suture anchor and debridement of the lesion (B) (F, fibula, P, peroneus tendon).

Suspecting suture anchor irritation of the peroneus tendon, we proceeded to surgery. We found a granulomatous mass-like lesion around the peroneus tendon at the site of tenderness (Fig. 4) and the muscle portion of the peroneus tendon was affected by the protruded superior suture anchor during motion. The suture anchor at the posterior distal fibula area, which irritated the peroneus tendon, was removed and the granulomatous lesion was debrided. We ensured that the peroneus tendon glided in the peroneal groove without impingement. There were no other lesions that might cause pain at the tender site. A biopsy of the excised mass revealed chronic inflammation with granulation tissue formation. At the 3-month follow-up, the patient was almost asymptomatic and had a nearly full range of motion (ROM). She had no complications or recurrent symptoms. At the 1-year follow-up, the Ankle-Hindfoot scale of the AOFAS score had improved to 97 and the VAS to 0 points. The patient had a satisfactory clinical outcome and resumed daily functional activities with no discomfort.

## 3 Discussion

Arthroscopic MBP is a good procedure for lateral ankle instability and has considerable benefits in terms of soft tissue dissection and recovery time, and outcomes comparable to open MBP.^[13,16,17,19]^ Yeo et al found no differences in the clinical or radiological outcomes between all-inside MBP and open MBP for the treatment of lateral ankle instability at the 1-year follow-up.^[13]^

However, various complications of arthroscopic MBP have been reported with the increasing popularity of arthroscopic MBP, including superficial peroneal nerve neuritis, sural nerve neuritis, portal site irritation, decreased ROM, residual or recurrent lateral ankle instability, and prominent asymptomatic suture knots.^[4,18]^ Symptomatic suture knot irritation led to the development of the knotless suture anchor.^[12,13,15,17]^ We use knotless suture anchors for arthroscopic MBP and have had no suture knot-related complications.

Some studies have reported peroneal tendon complications after application of a plate for lateral malleolar fractures.^[20]^ Ahn et al described peroneal tendinopathy after application of a posterior anti-glide plate for the repair of lateral malleolar fractures.^[21]^ They reported that the incidence of clinical peroneal tendinopathy was 4.29% (3 of 70) after placement of a posterior anti-glide plate for fibular malleolar fractures.

Similar to the plate for lateral malleolus fractures, when undergoing an arthroscopic MBP using suture anchors, a suture anchor that penetrates distal fibula can rarely cause peroneal tendinopathy. There is also a possibility of injuring the peroneal tendon with the drill while reaming for suture anchors.

Many other conditions can cause pain or discomfort similar to peroneal tendinopathy, including lateral ankle ligament sprains, lateral talar process fractures, ankle capsular impingement syndrome, distal tibiofibular syndesmotic ligament injury, lateral osteochondral lesions of the talus, subtalar joint pathology, os trigonum syndrome, sural neuropathy, and anterior process fractures of the calcaneus.^[21,22]^

Therefore, a careful examination is needed for an accurate diagnosis. If there is swelling, tenderness, or pain along the peroneal tendon, and if the pain becomes worse with the pain provocation test, or when the peroneal muscle power decreases, peroneal tendinopathy should be suspected.^[21]^

The drill hole for suture anchors is typically directed from anterior to posterior, perpendicular to the anterior surface of the fibula, and parallel to the plantar plane and plane of the lateral gutter to reproduce the native insertion of the ATFL.^[3,19,23,24]^ In our case, the anchor was inserted past the far cortex of the fibula and the muscle portion of the peroneus tendon was stimulated because the suture anchor was not inserted perpendicular to the fibula. We believe that the location and length of the suture anchor inserted are important for preventing peroneal tendinopathy when doing arthroscopic MBP using a suture anchor.

## 4 Conclusion

Many arthroscopic MBP techniques have been introduced, but the proper position and location of the suture anchor according to the shape of the distal fibula has not been established. Further radiographic studies analyzing the appropriate depth and position of suture anchors using 3-dimensional computed tomography are needed to standardize the procedure and reduce complications.

## Acknowledgment

The authors thank the Soonchunhyang University Research Fund for support.

## Author contributions

**Conceptualization:** Young Koo Lee.

**Project administration:** Sung Hun Won.

**Resources:** Hyun Kwon Kim.

**Software:** Chang Hyun Kim.

**Supervision:** Hong Seop Lee, Aeli Ryu, Woo Jong Kim.

**Writing – original draft:** Whi Je Cho, Woo Jong Kim.

**Writing – review and editing:** Woo Jong Kim.

Woo Jong Kim orcid: 0000-0002-4579-1008.
